# Productive Infection of Human Breast Cancer Cell Lines with Human Cytomegalovirus (HCMV)

**DOI:** 10.3390/pathogens10060641

**Published:** 2021-05-23

**Authors:** Kaitlin M. Branch, Erica C. Garcia, Yin Maggie Chen, Matthew McGregor, Mikayla Min, Rachel Prosser, Natalia Whitney, Juliet V. Spencer

**Affiliations:** 1 Department of Biology, University of San Francisco, San Francisco, CA 94117, USA; kmbranch@usfca.edu (K.M.B.); ychen254@dons.usfca.edu (Y.M.C.); mmcgregor2@dons.usfca.edu (M.M.); mkmin@dons.usfca.edu (M.M.); rmprosser@dons.usfca.edu (R.P.); nbwhitney@dons.usfca.edu (N.W.); 2 Department of Biology, Texas Woman’s University, Denton, TX 76204, USA; Egarcia48@twu.edu

**Keywords:** breast cancer, cancer, cytomegalovirus, HCMV

## Abstract

Breast cancer is the leading cause of cancer deaths among women worldwide. There are many known risk factors for breast cancer, but the role of infectious disease remains unclear. Human cytomegalovirus (HCMV) is a widespread herpesvirus that usually causes little disease. Because HCMV has been detected in breast tumor biopsy samples and is frequently transmitted via human breast milk, we investigated HCMV replication in breast tumor cells. Four human breast cancer cell lines with different expression profiles for the key diagnostic markers of the estrogen receptor (ER), progesterone receptor (PR), and human epidermal growth factor receptor 2 (HER2), were infected with a bacterial artificial chromosome-derived HCMV clinical strain TB40/E tagged with green fluorescent protein (GFP). Fluorescence microscopy confirmed that all four breast cancer cell lines supported virus entry. RNA was isolated from infected cells and the expression of immediate early (UL123), early (UL54), and late (UL111A) genes was confirmed using PCR. Viral proteins were detected by immunoblotting, and viral progeny were produced during the infection of breast tumor cells, as evidenced by subsequent infection of fibroblasts with culture supernatants. These results demonstrate that breast tumor cells support productive HCMV infection and could indicate that HCMV replication may play a role in breast cancer progression.

## 1. Introduction

Virus infection is associated with 15% to 20% of cancers worldwide [1]. Human papillomavirus is strongly linked to cervical cancer, and hepatitis B and C viruses are linked to hepatocellular carcinoma [2]. In contrast, the role of infectious agents in breast cancer is poorly understood [1,3]. A variety of viral infections have been implicated in breast cancer, including bovine leukemia virus [4,5], human mammary tumor virus [6], human papillomavirus [7], Epstein–Barr virus (EBV) [8,9,10], and human cytomegalovirus (HCMV) [11,12]. While there is no clear link between these viruses and breast cancer, molecular and epidemiological evidence suggests some association between HCMV and breast cancer [11,12,13,14,15,16,17,18].

HCMV is a member of the *Herpesviridae* family that is widespread in the population and can establish lifelong persistent or latent infection. Most infections are subclinical, and serious HCMV disease typically occurs only in immune-compromised individuals. HCMV is not considered an oncogenic virus [13,19]. However, HCMV infection can promote many classic hallmarks of cancer [20], such as cell cycle dysregulation, inhibition of apoptosis, and increased migration and invasion [21,22]. Viral DNA and proteins have been found in tumors, and the infection may contribute to tumor development or progression [19]. HCMV is transmitted in body fluids, including breast milk [23,24]. One study detected HCMV immediate early 1 (IE1) protein in breast glandular epithelial cells from most patients (97%) with ductal carcinoma in situ or infiltrating ductal carcinoma [11]. Another study found both HCMV IE1 and late proteins in metastatic tumor cells of breast cancer specimens (73/73 total) and detected viral DNA in 12/12 samples [12]. Additionally, in patients with early-onset breast cancer, CMV IgG antibody titers were significantly associated with breast cancer, whereas no association was found with EBV IgG titers [16,25].

While many studies have investigated HCMV DNA and proteins in patient biopsy samples, few have examined virus infection in breast cancer cells in vitro. Costa et al. infected MCF-7 and MDA-MB-231 breast cancer cells with a clinical strain of HCMV and observed induction of cellular cyclooxygenase-2 and 5-lipoxygenase protein expression [26]. However, aside from detection of IE1 by PCR to confirm infection, HCMV gene expression and replication were not examined. Oberstein and Shenk infected MDA-MB-231 and SUM1315MO2 breast cancer cell lines and noted that, while many cells expressed IE1, only a subset expressed late genes, suggesting stalled or delayed virus replication [27]. High expression of IE1/2 transcripts in HCMV-infected MFC-7, MDA-MB-361, MDA-MB-231, BT-549, and SUM1315MO2 cells at 24 hpi was also reported by Nogalski and Shenk [28]. Although productive infection was not observed, the proliferation and motility of the breast cancer cell lines was enhanced following HCMV infection.

MCF-7 and MDA-MB-231 are two of the most common breast cancer cell lines used for in vitro studies, and they represent distinct types of breast tumors. MDA-MB-231 cells are considered triple negative because they lack overexpression of the estrogen receptor (ER), progesterone receptor (PR), and human epidermal growth factor receptor 2 (HER2). Triple-negative breast tumors are among the most severe clinical subtype and extremely difficult to treat since many therapeutics target these receptors. In contrast, MCF-7 cells overexpress ER and PR, but not HER2, and they represent the Luminal A subtype, one of the most diagnosed forms of breast cancer. Luminal A breast tumors are generally less invasive but more responsive to treatment (60). In this study, we infected breast cancer cell lines with HCMV and evaluated viral gene expression, protein levels, and generation of infectious progeny. The results demonstrate that breast cancer cells support limited but productive HCMV infection.

## 2. Results

**Figure 1:**
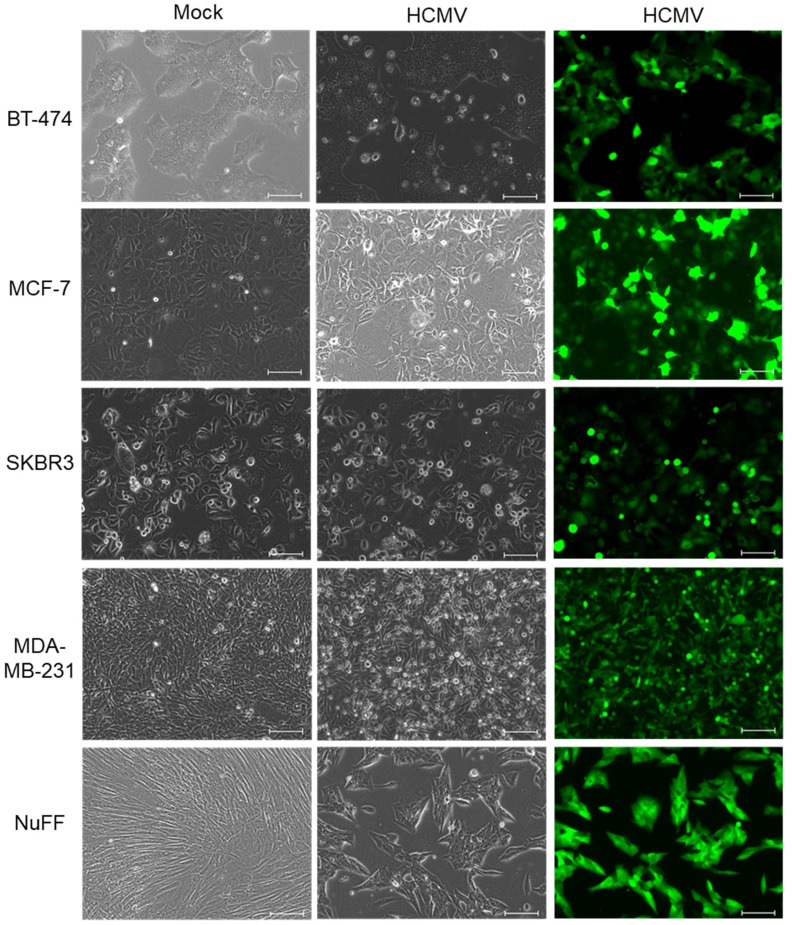
HCMV infection of breast cancer cells. Monolayer cultures of the indicated cell types were infected with HCMV strain TB40/E-GFP for 72 h (MOI = 1). Images were captured using bright field and fluorescence microscopy. Scale bar = 100 µm.

To investigate whether human breast cancer cells could be infected with HCMV, four different cell cancer cell lines were used. Since breast tumors vary in the expression of key diagnostic receptors (ER, PR, HER2), we selected cell lines that each expressed a different combination of these receptors. BT-474 (triple positive), MCF-7 (ER^+^PR^+^), SKBR3 (HER2^+^), and MDA-MB-231 (triple negative) cells were infected with the HCMV clinical strain TB40/E-GFP and then monitored for signs of infection for 72 h. Neonatal foreskin fibroblast (NuFF) cells, which are readily infected by HCMV, were used as a positive control. Fluorescence microscopy revealed that all four cancer cell lines and the NuFF cells exhibited green fluorescence, confirming that virus infection with HCMV strain TB40/E-GFP had occurred (Figure 1). The rate of infection was not significantly different between the five cell types, as indicated by the comparable number of GFP-positive cells in each culture. Mock-infected cells did not show any green fluorescence, consistent with the fact that they were not exposed to the virus. Bright field microscopy revealed that morphological changes were evident in infected cells compared to mock-infected cells, with cytopathic effects (CPE) being the most pronounced in NuFF cells. These results demonstrate that breast cancer cells are permissive for HCMV entry independent of ER, PR, or HER2 status.

**Figure 2:**
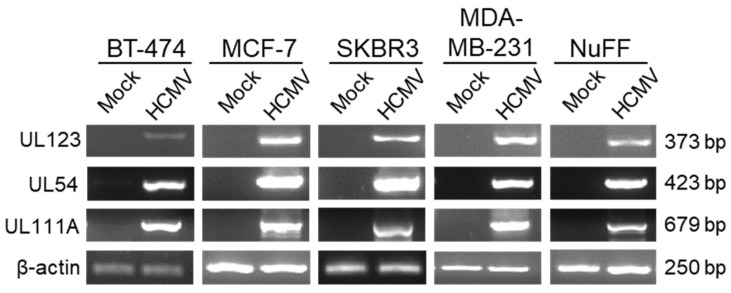
Breast cancer cells support viral gene expression. RNA was harvested from mock- or HCMV-infected cells at 72 hpi, reverse-transcribed to cDNA, and PCR was performed using gene-specific primers. Resulting bands were visualized by agarose gel electrophoresis.

Although the GFP gene expression was evident following infection of breast cancer cells, this gene is under control of an SV40 promoter and does not represent HCMV gene expression. We next examined the expression of representative HCMV genes. Cells were infected and after 72 h, RNA was harvested, reverse-transcribed to cDNA, and viral gene expression was evaluated by PCR (Figure 2). Because HCMV gene expression is a temporal cascade, we evaluated one representative immediate early (UL123), early (UL54), and late (UL111A) gene to determine if infection proceeded through all stages in breast cancer cells. Expression of UL123 (immediate early 1 gene) was seen in all four cancer cell lines and in NuFF cells. In addition, UL54, which encodes the viral DNA polymerase, and UL111A, which encodes the viral IL-10 ortholog cmvIL-10, could also be detected in each cell type. Cellular β-actin served as a positive control and was expressed in both mock- and HCMV-infected cells. These results indicate that breast cancer cells are permissive for HCMV replication.

**Figure 3:**
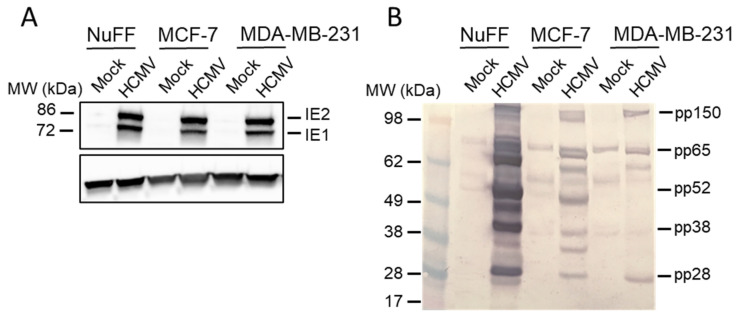
Viral proteins are produced in breast cancer cells. Cells were mock-infected or infected with TB40/E-GFP (MOI = 1). After 96 h, cell lysates were prepared, separated via SDS-PAGE, and immunoblotted using (**A**) antibodies directed against IE1/2 proteins, or (**B**) serum from a HCMV-seropositive donor. Representative viral proteins are indicated.

To evaluate the presence of viral proteins in HCMV-infected breast cancer cell lines, MCF-7 and MDA-MB-231 cells were harvested at 72 hpi and lysates were prepared. HCMV-infected NuFF cell lysates served as a positive control for infection. All lysates were separated by SDS-PAGE and immunoblotted with serum from an HCMV-positive donor (Figure 3). As expected, NuFF cells showed the greatest evidence of infection with a distinct banding pattern that was not present in mock-infected cells. Prominent bands at 28, 38, 52, 65, and 150 kD correspond to five viral proteins known to elicit strong humoral responses in most seropositive individuals [29,30,31]. These proteins include tegument proteins pp150 (UL32 gene product), pp65 (UL83), and pp28 (UL99), as well as DNA polymerase processivity factor pp52 (UL44) and viral assembly protein pp38 (UL80a). A comparable banding pattern was detected in HCMV-infected MCF-7 and MDA-MB-231 breast cancer cells but not in mock-infected cells, indicating that breast cancer cells support virus replication. Only faint bands for pp52 and pp38 were detected in MDA-MB-231 cells, which could reflect less efficient virus replication or slightly delayed replication kinetics in these cells.

**Figure 4:**
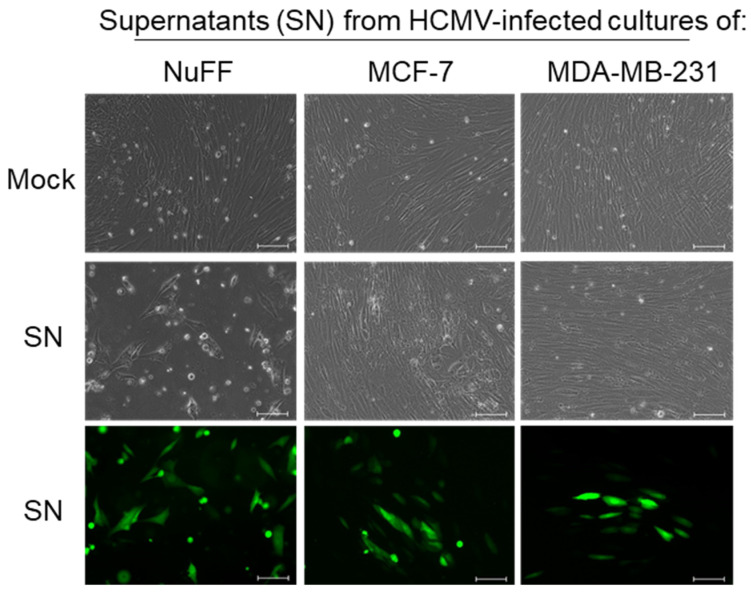
Breast cancer cells produce infectious progeny. Monolayer cultures of NuFF cells were treated with supernatants (SN) that had been harvested from mock- or HCMV-infected NuFF, MCF-7, or MDA-MB-231 cultures at 96 hpi. Cultures were examined by bright field and fluorescence microscopy 96 h after treatment with SN. Scale bar = 100 µm.

Finally, we wanted to determine whether infected breast cancer cells produced infectious progeny virions. NuFF, MCF-7, and MDA-MB-231 cells were infected with HCMV TB40/E-GFP, and after 96 h, supernatants were harvested and transferred to fresh monolayer cultures of NuFF cells. These NuFF cells were monitored for evidence of virus infection by bright field and fluorescence microscopy. As expected, HCMV infection of NuFF cells resulted in infectious virus production, and progeny virions from the NuFF supernatant infected the fresh NuFF monolayers (Figure 4), indicated by the presence of GFP-expressing cells. In addition, NuFF cells that received supernatants from MCF-7 and MDA-MB-231 cultures also expressed GFP and showed signs of infection. These results demonstrate that supernatants from HCMV-infected breast cancer cells contain infectious progeny virus, suggesting that breast cancer cells can be productively infected with HCMV.

## 3. Discussion

While HCMV DNA and proteins have been detected in breast tumor biopsy samples [11,12,18], virus replication in breast cancer cells has not previously been extensively examined. We found that four distinct types of breast cancer cell lines, each with a different ER/PR/HER2 profile, all support HCMV entry. We documented virus entry (Figure 1) and viral gene expression (Figure 2) in all four cell lines, and we demonstrate viral protein synthesis (Figure 3) and production of infectious viral progeny (Figure 4) for two cell lines. Virus replication was independent of hormone receptor status and was observed in MCF-7 cells, which overexpress ER as well as triple-negative MDA-MB-231 cells. These results suggest that breast cancer cells can be productively infected with HCMV.

Previous studies have found that HCMV infection in cancer cells is delayed or abortive. Oberstein and Shenk reported that despite expression of IE1 in HCMV-infected MDA-MB-231 cells, few cells expressed the late gene UL83 [27]. In those studies, expression of UL83 was observed only in cells infected with TB40/E virus stocks propagated in epithelial cells at an MOI of 10. In contrast, we used a TB40/E virus propagated on fibroblasts at an MOI of 1 and observed expression of early and late genes UL54 and UL111A (Figure 2). This discrepancy warrants further investigation. Because we analyzed RNA extracted from the whole culture, we cannot rule out the possibility that only a small subset of cells was expressing the early and late genes. Another possible explanation is that our virus titers were off by a factor of 10, although this seems unlikely. We found that supernatants from infected breast cancer cells contained the infectious virus after 96 h (Figure 4), indicating that at least some of the cells supported the full replication cycle and released progeny virions. Interestingly, we observed lower levels of several viral proteins in HCMV-infected MDA-MB-231 cells compared to MCF-7 cells, suggesting that the efficiency of viral protein expression may inversely correlate with the metastatic phenotype of breast cancer cells, in agreement with the findings of Oberstein and Shenk [27].

Considering that HCMV is transmitted in breast milk [23,24,32], virus replication in breast tumor cells is not surprising. For breast milk to contain the infectious virus, there must be productive HCMV infection in breast tissue. Moreover, HCMV proteins have been detected in normal breast tissue as well as in tumor biopsy samples [11]. HCMV can infect a range of cell types, including monocytes, macrophages, epithelial cells, endothelial cells, smooth muscle cells, and fibroblasts [33,34]. Fibroblasts were used as a positive control for these experiments since they are known to support robust HCMV replication in vitro. Fibroblasts have a distinct morphology, and cytopathic effects (CPE) due to HCMV infection are pronounced (Figure 1 and Figure 4). In cultures of HCMV-infected fibroblasts, we saw widespread cytopathic effects followed by cell death. In contrast, we did not observe dramatic changes in cell morphology in HCMV-infected breast cancer cell cultures. Additional work is needed to evaluate the impact of HCMV infection on breast cancer cell physiology, including examining the growth rate of infected breast cancer cells and exploring how they can retain normal cell morphology but also produce the infectious virus. For future experiments, we would use an epithelial cell line known to support HCMV replication as the positive control, such as adult retinal pigment epithelial cells (ARPE-19) or normal breast epithelial cells.

It is unclear whether HCMV infection directly impacts hormone receptor levels. Rahbar and colleagues examined infiltrating breast cancer (n = 62) and ductal carcinoma in situ (DCIS, n = 19) specimens and detected HCMV IE protein in all tissue specimens examined [35]. HCMV late (LA) protein was detected in 74% of the infiltrating breast cancers and in 47% of the DCIS specimens. Based on the percentage of cells expressing HCMV proteins, the samples were categorized as high-grade (>50%) or low-grade (<50%) HCMV infection. Interestingly, high-grade HCMV-IE expression was significantly associated with lower levels of ER and PR expression [35]. However, because the specimens were fixed biopsy samples, we do not know if HCMV infection caused the decrease in ER and PR levels. Kumar et al. demonstrated that HCMV infection of human mammary epithelial cells (HMECs) resulted in activation of oncogenic signaling pathways, led to colony formation in soft agar, and resulted in tumor formation when infected HMECs were injected into immunodeficient mice [36]. Immunohistochemistry of the HCMV-infected HMEC tumors indicated that they were triple negative for ER, PR, and HER2; however, receptor levels before and after infection were not evaluated. Additional work is needed to evaluate the effects of HCMV infection on ER and PR levels in breast cancer cells in culture. If HCMV infection does cause downregulation of ER and/or PR levels, this could impair patient responsiveness to standard ER-based treatments such as tamoxifen and letrozole.

HCMV has also been implicated in development of metastases. Most breast cancer deaths result not from local complications of the primary tumor, but rather from the development of metastases and the malignant spread of the tumor throughout the body. Taher et al. reported detection of HCMV via PCR in brain metastases of women with primary breast tumors [14]. In a recent in vivo study, breast cancer cells were injected into the mammary fat pad of mice that had been infected with murine CMV (MCMV) for various time points (4 days, 11 days, or 10 weeks), representing active, intermediate, or latent infection. While infection overall did not impact primary tumor size, mice with latent MCMV infection had more vascularized mammary tumors and a larger number of lung metastases compared to mice infected with the UV-inactivated virus [17]. An increase in lung metastases was not observed in mice with active or intermediate MCMV infection, suggesting that latency-associated genes may contribute to enhanced development of metastases. Additional work is needed to understand the impact of HCMV on metastasis formation in patients with breast cancer and whether antiviral treatments could improve patient outcomes.

## 4. Materials and Methods

### 4.1. Cells and Viruses

BT474, MCF-7, SKBR3, and MDA-MB-231 human breast cancer cells were obtained from the American Type Culture Collection (Manassas, VA, USA). Neonatal human foreskin fibroblasts (NuFFs) were obtained from Global Stem Cell Group (Miami Lakes, FL, USA). All cells were grown in Dulbecco’s modified Eagle’s medium (DMEM) containing sodium pyruvate, L-glutamine, and 4.5 g/L glucose, supplemented with 10% fetal bovine serum (FBS), non-essential amino acids (NEAA), and 1M HEPES buffer, and maintained at 37 °C in a humidified chamber with 5% CO_2_. Virus stocks of bacterial artificial chromosome (BAC)-derived clinical strain HCMV TB40/E-GFP [37,38] were prepared on confluent NuFF monolayers. This virus was constructed using recombineering methodology to insert a cassette containing GFP controlled by the SV40 promoter in the intergenic region between the HCMV US34 and TRS-1 ORFs as previously described [38]. Virus titers were determined by the 50% tissue culture infectious dose (TCID_50_) method. For infection experiments, cells were seeded into 10 cm^2^ tissue culture dishes at a density of 1.0 × 10^6^ cells/dish. The next day cells were infected at a multiplicity of infection (MOI) of 1 by adding TB40/E-GFP virus to 4 mL of infection medium (DMEM containing sodium pyruvate, L-glutamine, and 4.5 g/L glucose, and supplemented with 10% fetal calf serum, non-essential amino acids (NEAA), and 1M HEPES buffer). HCMV-infected cells were treated with virus inoculum while mock-infected cells received infection medium only for 90 min at 37 °C. After 90 min the inoculum was removed, cells were washed two times with sterile PBS, and fresh infection medium was added to a final volume of 8 mL. Infections were monitored daily via bright field and fluorescence microscopy for 72 h.

### 4.2. Reverse Transcription Polymerase Chain Reaction (RT-PCR)

At 72 h post-infection (hpi), mock- and HCMV-infected cells were harvested via trypsinization. RNA was extracted using the RNeasy kit (Qiagen, Carlsbad, CA, USA) and reverse-transcribed to cDNA using the iScript cDNA synthesis kit (Bio-Rad, Hercules, CA, USA) according to the manufacturer’s instructions. The polymerase chain reaction (PCR) was used to detect expression of the HCMV UL123 (immediate-early), UL54 (early), and UL111A (late) genes. The cellular gene β-Actin was used as a loading control. Primers pairs for detection of each gene were as follows: UL123 (GGTCACTAGTGACGCTTGTATGATGACCATGTACCGA and GATAGTCGCGGGTACAGGGGACTCT), UL54 (CGGCTACAGTATCTGCGTCA and AGCCACCAGGTCAGAGACAT), UL111A (GGGGGATCCATGCTGTCGGTGATGG and CTTTCTCGAGTGCAGATAC), and β-actin (ATTAAGGAGAAGCTGTGCTACG and TGTTGGCGTACAGGTCTTTG). Each RT-PCR reaction contained a cDNA template (500 ng), primers, dNTP mix, Ex-Taq buffer, and Ex-Taq polymerase (Clontech, Mountain View, CA, USA) in a final volume of 25 µl. Negative controls lacking the cDNA template were also performed. The PCR protocol was 95 °C for 1 min, 94 °C for 1 min, 60 °C for 1 min, 72 °C for 1 min, for 35 cycles and then 72 °C for 10 min. PCR products were visualized on a 2% agarose gel and imaged using the Bio-Rad ChemiDoc XP imaging system.

### 4.3. Immunoblotting

At 72 hpi breast tumor cells and NuFF cells were harvested and pelleted by centrifugation at 1000 RPM for 5 min at room temperature. Cell pellets were washed in 1 mL of ice-cold phosphate buffered saline (PBS), re-pelleted, and then resuspended in 100 µL cell lysis buffer (150 mM NaCl, 20 mM HEPES, 0.5% Triton X-100, 1 mM NaVO_4_ 1 mM EDTA, and 0.1% NaN_3_) and sonicated to further disrupt cell membranes. Cells were then centrifuged for 15 min at 14,000 RPM and 4 °C, and the supernatant was collected, combined with sample loading dye and DTT, and then heated at 70 °C for 10 min. Samples were separated on 4–12% Bis-Tris protein gels with 3-(N-morpholino) propane sulfonic acid (MOPS) buffer and transferred to nitrocellulose membranes for immunoblotting. Next, membranes were blocked in a TBS-T/5% milk-blocking solution for 1 h at room temperature on a plate shaker. Antibodies directed against IE1/2 protein (Abcam, Cambridge, UK ab53495) were used at 1:1000 dilution. HCMV-positive patient serum, used in place of a commercial primary antibody, was heat-inactivated for 30 min at 56 °C and prepared at a 1:1500 dilution as described previously [39]. Nitrocellulose membranes were incubated with HCMV-positive patient serum overnight, shaking at 4 °C. The following day, after three TBS-T washes, goat anti-human polyclonal IgG (Santa Cruz Biotechnology, Dallas, TX, USA, sc-2454) conjugated to alkaline phosphatase (AP) was prepared at 1:1000. After three additional washes with TBS-T, and two washes with dH_2_0, and the addition of chromogenic substrate for 30–60 min for detection, membranes were washed with dH_2_O and analyzed.

### 4.4. Virus Infectivity Assay

At 96 hpi, supernatants (SN) were harvested from mock- and HCMV-infected MCF-7, MDA-MB-231, and NuFF cells and clarified by centrifugation at 1000 rpm for 5 min. SNs from one well of infected NuFF or breast cancer cells were used to directly infect a well of fibroblasts; SNs were not pooled. Approximately 2 mL of SN was added to each well of a 6-well tissue culture plate containing NuFF cells that had been seeded 48 h prior at a density of 2 × 10^5^ cells/well. SN-treated NuFF cells were placed in a humidified chamber at 37 °C with 5% CO_2_ for 90 min. After 90 min, inoculum was removed and cells were washed two times with sterile PBS to remove the inoculum. Fresh infection medium was added to each well for a total volume of 2 mL/well. Cells were monitored for signs of infection via fluorescence microscopy for seven days.

## Data Availability

Not applicable.
